# Cost-Effectiveness Analysis of Implantable Collamer Lens (ICL) Surgery Versus Contact Lens-Based Correction with Backup Spectacles in Young Adults—A Norwegian Societal Perspective

**DOI:** 10.3390/healthcare14193220

**Published:** 2026-09-30

**Authors:** Silvia N. W. Hertzberg, Ashraf Armia Balamoun, Filippo Confalonieri, Germán Roberto Bianchi, Robert Rejdak, Beata Eva Petrovski, Goran Petrovski

**Affiliations:** 1 Center for Eye Research and Innovative Diagnostics, Department of Ophthalmology, Oslo University Hospital, 0450 Oslo, Norway; swalekhwa@gmail.com (S.N.W.H.); b.e.petrovski@medisin.uio.no (B.E.P.); 2 Watany Eye Hospital, Cairo 16112, Egypt; ashrafarmia@gmail.com; 3 Ashraf Armia Eye Clinic, Cairo 12655, Egypt; 4 Department of Ophthalmology, Montecchi Hospital of Suzzara, 46029 Suzzara, Mantua, Italy; filippo.confalonieri01@gmail.com; 5 Dr Nano Eye Clinic, Olivos B1636CSS, Buenos Aires, Argentina; drbianchigerman@gmail.com; 6 Department of General and Pediatric Ophthalmology, Medical University of Lublin, 20-059 Lublin, Poland; robertrejdak@yahoo.com; 7 Institute for Clinical Medicine, University of Oslo, 0450 Oslo, Norway; 8 Department of Ophthalmology, University Hospital Centre, University of Split School of Medicine, 21000 Split, Croatia; 9 UKLOnetwork, University St. Kliment Ohridski-Bitola, 7000 Bitola, North Macedonia

**Keywords:** implantable collamer lens (ICL), contact lenses, cost-effectiveness analysis, quality-adjusted life-years, health economic modelling, refractive surgery

## Abstract

**Background**: Moderate-to-high refractive error generates long-term direct and indirect costs through corrective devices, complications, and productivity losses. The economic value of implantable collamer lens (ICL) surgery relative to contact lens (CL)-based correction with backup spectacles remains uncertain. **Objective**: To evaluate the 25-year cost-effectiveness of posterior chamber phakic ICL implantation versus CL-based correction across four refractive subgroups from a Norwegian societal perspective. **Methods**: A Markov cohort model followed patients aged 25–50 years with high myopia, high myopia with astigmatism, high hyperopia, or high hyperopia with astigmatism. Health states incorporated surgical complications, microbial keratitis (MK), cataract, explantation, and death. Direct medical and productivity costs were valued in 2024 Norwegian kroner (NOK), converted to United States dollars (USD), and subsequently reported in USD. Outcomes included quality-adjusted life-years (QALYs), incremental costs, incremental net monetary benefit (INMB), and cost-effectiveness probabilities at Norwegian severity-based thresholds (NOK 250,000–825,000/QALY). Deterministic and probabilistic sensitivity analyses were performed. **Results**: In the deterministic base case, ICL was less costly and more effective in all subgroups. Incremental QALY gains ranged from 0.049 to 0.464, and 25-year savings ranged from USD 1758 to USD 2277 per patient. At USD 27,253/QALY, INMB ranged from USD 3264 to USD 14,921. ICL produced approximately 5–6 additional cataract surgeries per 100 patients but avoided approximately 1.1–1.3 MK infections per 100 CL users and provided 0.6–0.9 additional years in the “Well” state. Cost-effectiveness probabilities ranged from 52.7% to 74.2% and were highest in the astigmatic subgroups. Utilities were the principal uncertainty drivers; the 52.7% probability observed for high hyperopia indicated limited robustness. **Conclusions**: Over 25 years, ICL may provide cost savings and quality-of-life gains compared with CL-based correction in appropriately selected patients. These findings depend on model assumptions and should not be interpreted as a lifetime evaluation.

## 1. Introduction

Refractive error, particularly moderate-to-high myopia, is a substantial global health challenge, with high myopia projected to increase markedly by 2050 [1]. European population data show an especially high prevalence of myopia among young adults [2]. Uncorrected or undercorrected refractive error causes major productivity losses [3,4,5], while continued contact lens wear incurs recurring costs and carries a small but clinically important risk of microbial keratitis [6,7].

For healthcare systems, the relevant question is whether a durable surgical intervention provides value for money over a prespecified time horizon compared with continued non-surgical management. ICL represents an upfront healthcare investment intended to yield future benefits through avoided device-related costs and gains in quality-adjusted life-years (QALYs). Patients with high myopia who received ICL have reported better vision-related quality of life than successful contact lens wearers [8], but economic findings are not automatically transferable between countries because prices, healthcare pathways, reimbursement policies, wages, and cost-effectiveness thresholds differ [9,10,11,12,13]. A Norwegian analysis is therefore warranted.

Unlike corneal-based procedures—including photorefractive keratectomy (PRK), laser-assisted in situ keratomileusis (LASIK), and keratorefractive lenticule extraction (KLEx)—ICL does not remove corneal stromal tissue and can correct a broad range of refractive errors. It is therefore an important option for selected patients with thin corneas or refractive errors outside the preferred range for corneal laser surgery. Reviews and long-term studies have reported predictable outcomes and high patient satisfaction, while identifying risks including endothelial cell loss, lens rotation or explantation, and cataract formation [14,15,16,17,18,19,20]. From a health technology assessment perspective, the objective is to estimate incremental costs and effects and determine whether the benefits of ICL justify its upfront and downstream costs.

Although patients using CLs and those receiving ICL may achieve similarly high contrast visual acuity, patient-reported outcomes indicate better vision-related quality of life after ICL implantation in patients with high myopia [8]. Longitudinal economic evaluations of permanent surgical correction versus continued CL-based correction remain scarce [9,10]. Existing evidence is not directly transferable to Norway because recurring device prices, travel costs, productivity valuation, and priority-setting thresholds are jurisdiction-specific.

Health technology assessment frameworks recommend explicit long-term modelling, uncertainty analysis, and transparent reporting for surgical interventions [21,22,23]. To the authors’ knowledge, no prior Norwegian evaluation integrated age-specific Markov transitions, subgroup analysis, and probabilistic uncertainty to assess ICL implantation from young adulthood to midlife. The objective was therefore to estimate the 25-year incremental costs and QALYs and the probability of cost-effectiveness of ICL implantation versus CL-based correction with backup spectacles across four refractive subgroups.

## 2. Methods

### 2.1. Study Design and Perspective

A cohort-based state-transition (Markov) model was developed to evaluate the cost-utility of ICL implantation compared with continued CL correction in adults aged 20–30 years with high refractive error in the Norwegian population. The base-case cohort entered the model at age 25 years, representing the midpoint of the eligible population, and was followed for 25 annual cycles (age 25–50 years). This time horizon was selected to capture the period during working-age adulthood in which the strategies were expected to differ most in costs, complication risk, productivity effects, and quality-of-life outcomes. Thus, in the primary analysis, no extrapolation beyond age 50 was performed. This analysis adopted a societal perspective, incorporating direct medical costs, direct non-medical costs, and productivity losses. A health-service payer perspective excludes travel and productivity effects and therefore narrows the estimated cost difference. Costs and quality-adjusted life-years (QALYs) were discounted at 3% annually. The model was implemented in R version 4.4.1 (R Foundation for Statistical Computing, Vienna, Austria), using base R matrix operations and the dplyr 1.1.4 and ggplot2 3.5.1 packages for data management and visualization. The analytic framework followed CHEERS 2022 reporting standards [23].

### 2.2. Population and Subgroups

The simulated cohort represented adults aged 20–30 years who were eligible for either ICL implantation or continued CL-based correction; the base case began at age 25 years (Table 1). High myopia was defined as a spherical equivalent of ≤−6.00 dioptres (D), consistent with the European Eye Epidemiology definition [2]. High hyperopia was defined as a spherical equivalent of ≥+5.00 D and clinically significant astigmatism as a cylinder magnitude of ≥2.00 D; these thresholds were selected to represent refractive errors for which lens-based correction is commonly considered [2,24,25]. Analyses were stratified across four modelled subgroups: high myopia, high myopia with astigmatism, high hyperopia, and high hyperopia with astigmatism. This was a model-based evaluation and did not enrol individual participants; participant-level demographics were therefore not collected. Each subgroup was modelled independently, with modifiers applied to utilities, costs, and transition probabilities.

### 2.3. Model Structure and Health States

A cohort-based state-transition (Markov) model was developed comprising mutually exclusive health states. These included successful ICL implantation without current complication; temporary ICL complications not requiring explantation; ICL explantation followed by spectacle correction; clinically significant cataract following ICL implantation; CL wearer with minor or frequent complications; acute CL-associated microbial keratitis; permanent visual loss following severe keratitis; glasses/spectacle correction; and death (an absorbing state).

Transition probabilities were implemented in age-specific annual transition matrices, with events assumed to occur at mid-cycle. The matrices were recalculated for each cycle. Modelled transitions included ICL explantation, non-explant ICL complications, cataract following ICL implantation, CL-associated microbial keratitis, permanent visual loss after severe keratitis, discontinuation of CL use and transition to spectacle correction, and death. After discontinuing CL use, patients entered the spectacle state and accrued the corresponding spectacle costs and utility. Age-specific all-cause mortality was obtained from Statistics Norway life tables. Transition rules and base-case probabilities are reported in Supplementary Tables S1 and S2.

Health-state utilities and transition probabilities were derived from peer-reviewed literature [6,8,14,15,16,17,26,27,28,29,30,31,32,33,34,35]. Utilities were assigned at the health-state level on a scale of 0–1. The utilities for ICL and CL ‘Well’ states were anchored to the observed quality-of-life difference between ICL recipients and successful CL wearers [8]. Where direct utility estimates were unavailable, visual acuity outcomes were mapped to time trade-off utilities using published valuation studies [26,36,37,38]. Base-case values, subgroup modifiers, complication rates, probability distributions, and sources are reported in Supplementary Table S2.

During each annual cycle,

QALY cycle = ∑ (state proportion × utility) × discount factor.

Cataract was modelled as an ICL-attributable adverse event in the base case. Background age-related cataract was excluded from the CL arm to isolate the excess surgical risk; this is conservative for ICL because no background cataract burden was assigned to the comparator. In a structural scenario, the same age-specific background cataract risk and treatment costs were added to both strategies. Because the added risk was identical and independent of treatment, it cancelled out in the incremental comparison and did not change incremental costs, QALYs, or the preferred strategy. Mild CL-related symptoms such as dry eye were incorporated into the CL ‘Well’ state through its utility and associated cost. The annual probability of CL discontinuation was 0.217, based on the mean dropout frequency reported by Pucker and Tichenor [27]; it was applied in each annual cycle, after which patients transitioned to spectacle correction. Hence, total QALYs represent the discounted sum of QALYs accrued across 25 annual cycles and can exceed 1 over the full time horizon.

Norwegian unit costs were obtained from Statistics Norway, the Norwegian Medical Products Agency, national reimbursement schedules, and publicly listed private-clinic prices. Where cost estimates varied, conservative averages were used. Costs were valued in 2024 Norwegian kroner (NOK) and converted to United States dollars (USD) at 1 NOK = USD 0.0991 (8 July 2025). Subsequent monetary results are reported in USD. The initial ICL cost included manifest and cycloplegic refraction, corneal topography/tomography and pachymetry, anterior chamber depth and white-to-white measurements, endothelial cell count, tonometry, dilated retinal examination, lens calculation, and bilateral surgery. Travel costs per visit represented a return journey and included the associated patient travel time, valued using the same national costing framework; outpatient clinical fees were accounted for separately. Primary spectacles were replaced every two years, whereas backup spectacles were replaced every three years because of their lower frequency of use. The higher unit cost assigned to backup spectacles reflected prescription lenses suitable for the high-refractive-error subgroups rather than a difference in replacement frequency.

Norway unit costs were used as outlined in various cost databases (Statistics Norway, NoMA, private clinic pricing), and in cases of varied costs, conservative average estimates were used. All costs were expressed in 2024 NOK and converted to USD using the 8 July 2025 rate (1 NOK= 0.0991 USD [Norges Bank]) (Table 2).

Costs were applied as one-time initial costs at model entry (i.e., ICL surgery cost, ICL follow-up year 1, and CL fitting costs) and as annual state-based costs thereafter. Annual state costs were adjusted for each subgroup using multiplicative factors, and Gamma distributions were used for sampling.

Each subgroup was simulated independently to estimate total discounted costs and QALYs, incremental costs and incremental QALYs, and incremental cost-effectiveness ratios (ICER, cost/QALY) of ICL versus the contact lenses. Given the limitations of ICER interpretation in cases of dominance or extended dominance, incremental net monetary benefit (INMB) was also calculated. The willingness-to-pay (WTP) threshold was set at USD 27,253 (275,000 NOK)/QALY, with additional reference thresholds reflecting Norwegian priority levels (Norwegian Directorate of Health 2024).

Deterministic one-way sensitivity analysis explored parameter uncertainty using INMB as the outcome. Various parameters were varied at different values, and these included ICL surgery cost and follow-up costs (±15%), ICL and CL utilities (±0.04 additive shift), CL microbial keratitis and ICL revision probabilities (±30%), CL dropout (±50%), and societal salary, backup glasses, and cataract costs (all ±20%). These were scenario bounds, not confidence intervals. They were intentionally wider for uncertain event probabilities and narrower for tariff-based costs. Exact bounds and distributional assumptions are reported in Supplementary Table S2. Results were visualized as tornado plots displaying both positive and negative INMB deviations from the base case.

### 2.4. Probabilistic Sensitivity Analysis

A Monte Carlo simulation with 5000 iterations quantified joint parameter uncertainty. For each iteration, the model drew random samples for utilities (Beta), costs (Gamma), and risk multipliers (Lognormal). Age-specific transition matrices were perturbed accordingly, and both ICL and CL cohorts were simulated. Each iteration yielded incremental costs (ΔCost) and incremental QALYs (ΔQALY).

Outputs included cost-effectiveness planes, cost-effectiveness acceptability curves (CEACs), cost-effectiveness acceptability frontiers (CEAFs), and population-weighted CEACs. ICL was deemed cost-effective when INMB = (WTP × ΔQALY − ΔCost) > 0. CEACs and CEAFs summarized the probability of cost-effectiveness across thresholds from USD 0 to USD 84,000 per QALY.

Assumptions:

- Subgroup modifiers adequately capture clinical heterogeneity.
- Lognormal distributions appropriately reflect uncertainty in multiplicative risk adjustments.
- Productivity loss estimates reflect average working-age earnings.
- Cataract incidence was assumed to be exclusively an ICL-attributable adverse event in the modelled population.

### 2.5. Model Validation

Internal validation confirmed that every transition-matrix row summed to 1, cohort membership was conserved across cycles, absorbing-state behaviour was correct, and deterministic calculations reproduced the tabulated results. Face validity was assessed by the clinical and health-economic author group through review of the state structure, event ordering, and outcome direction. External validation was limited by the absence of a directly comparable Norwegian ICL economic evaluation; modelled event rates were checked against published ICL and CL complication ranges [6,14,15,16,17]. Extensive deterministic, probabilistic, and threshold analyses were performed to test model robustness.

## 3. Results

### 3.1. Deterministic Base-Case Analysis

Across all four refractive subgroups, implantation of a posterior chamber phakic intraocular lens was both less costly and more effective than a 25-year period of CL-based correction, indicating strict dominance in every subgroup (Table 3).

### 3.2. High Myopia

ICL generated 15.815 QALYs compared with 15.598 QALYs for CL, yielding an incremental gain of 0.217 QALYs. Total costs over the 25-year horizon were USD 11,013 (NOK 111,126) and USD 12,770 (NOK 128,864) for ICL and CL, respectively, corresponding to cost savings of USD 1758 (NOK 17,737) per patient. The incremental net monetary benefit (INMB), evaluated at a willingness-to-pay (WTP) threshold of USD 27,253 (NOK 275,000) per QALY, was USD 7662 (NOK 77,318). ICL therefore dominated CL-based correction.

While High Myopia with astigmatism, ICL produced 15.971 QALYs versus 15.507 QALYs for CL (ΔQALY 0.464). Total costs over the 25-year horizon were USD 14,397 (NOK 145,273) and USD 16,670 (NOK 168,212) for ICL and CL, respectively, resulting in cost savings of USD 2273 (NOK 22,939) per patient. The INMB was USD 14,921 (NOK 150,566). ICL therefore strongly dominated CL-based correction in this subgroup.

### 3.3. High Hyperopia

ICL yielded 15.647 QALYs compared with 15.598 QALYs for CL (ΔQALY 0.049). Total costs were USD 12,137 (NOK 122,469) and USD 14,067 (NOK 141,944) for ICL and CL, respectively, corresponding to savings of USD 1930 (NOK 19,476) per patient. The INMB was USD 3264 (NOK 32,939). ICL remained cost-saving and more effective.

Among those with astigmatism, ICL generated 15.892 QALYs versus 15.507 QALYs for CL (ΔQALY 0.385). Costs were USD 14,370 (NOK 145,005) for ICL and USD 16,647 (NOK 167,982) for CL, producing savings of USD 2277 (NOK 22,977) per patient. The INMB was USD 12,762 (NOK 128,780). ICL dominated CL.

### 3.4. One-Way Sensitivity Analysis

One-way sensitivity analysis identified utility parameters as the primary drivers of economic outcomes across all subgroups. Key Drivers (ranked by INMB range) were identified as utility of the ICL option, utility of the CL option, ICL surgery cost, ICL ongoing cost, and ICL revision probability. ICL revision probability was the fifth-ranked driver in all subgroups except high hyperopia, for which CL complication probability was the fifth-ranked driver (Supplementary Figure S3). ICL utility exhibited the widest INMB range (up to ~USD 3600/NOK 36,000), followed by CL utility (~USD 1970/NOK 19,750). Cost parameters exerted substantially smaller influence within the specified ranges.

### 3.5. Threshold Sensitivity

Across all subgroups, ICL remained cost-effective across the Norwegian priority-setting thresholds, including low severity, moderate severity, and high severity, although the probability of cost-effectiveness decreased with increasing thresholds, except for high hyperopia. High Myopia + Astigmatism and High Hyperopia + Astigmatism had the highest probabilities of being cost-effective at all thresholds (Table 4).

Markov trace analysis demonstrated that the majority of ICL-treated patients remained in the “Well_ICL” health state over the 25-year horizon. Revision, cataract, and explantation states occurred infrequently and did not materially offset the overall QALY gains (Supplementary Figure S1). In contrast, CL cohorts experienced persistent exposure to the long-term risk of microbial keratitis and post-infectious visual loss. Mortality trajectories were comparable across strategies, reflecting similar background age-dependent mortality assumptions (Supplementary Figure S1).

### 3.6. Probabilistic Sensitivity Analysis Results

Probabilistic sensitivity analysis (PSA) used 5000 Monte Carlo simulations. The results supported the direction of the deterministic findings but showed substantial decision uncertainty in some subgroups, particularly high hyperopia.

This 25-year societal-perspective evaluation found that ICL was less costly and more effective than CL-based correction in the deterministic base case across four refractive subgroups. Per-patient savings ranged from approximately USD 1758 to USD 2277, and incremental QALYs from 0.049 to 0.464. Probabilistic results were most favourable in the astigmatic subgroups but were less certain for high hyperopia, for which the base-case probability of cost-effectiveness was 52.7%.

Expected value of perfect information (EVPI) was explored during model development but was not retained as a prespecified reported outcome because population size and effective decision lifetime—both required for population EVPI—were not defined consistently. EVPI has therefore been removed from the final Methods and Results rather than reported incompletely.

### 3.7. Cost-Effectiveness Planes (CE)

**Figure 1:**
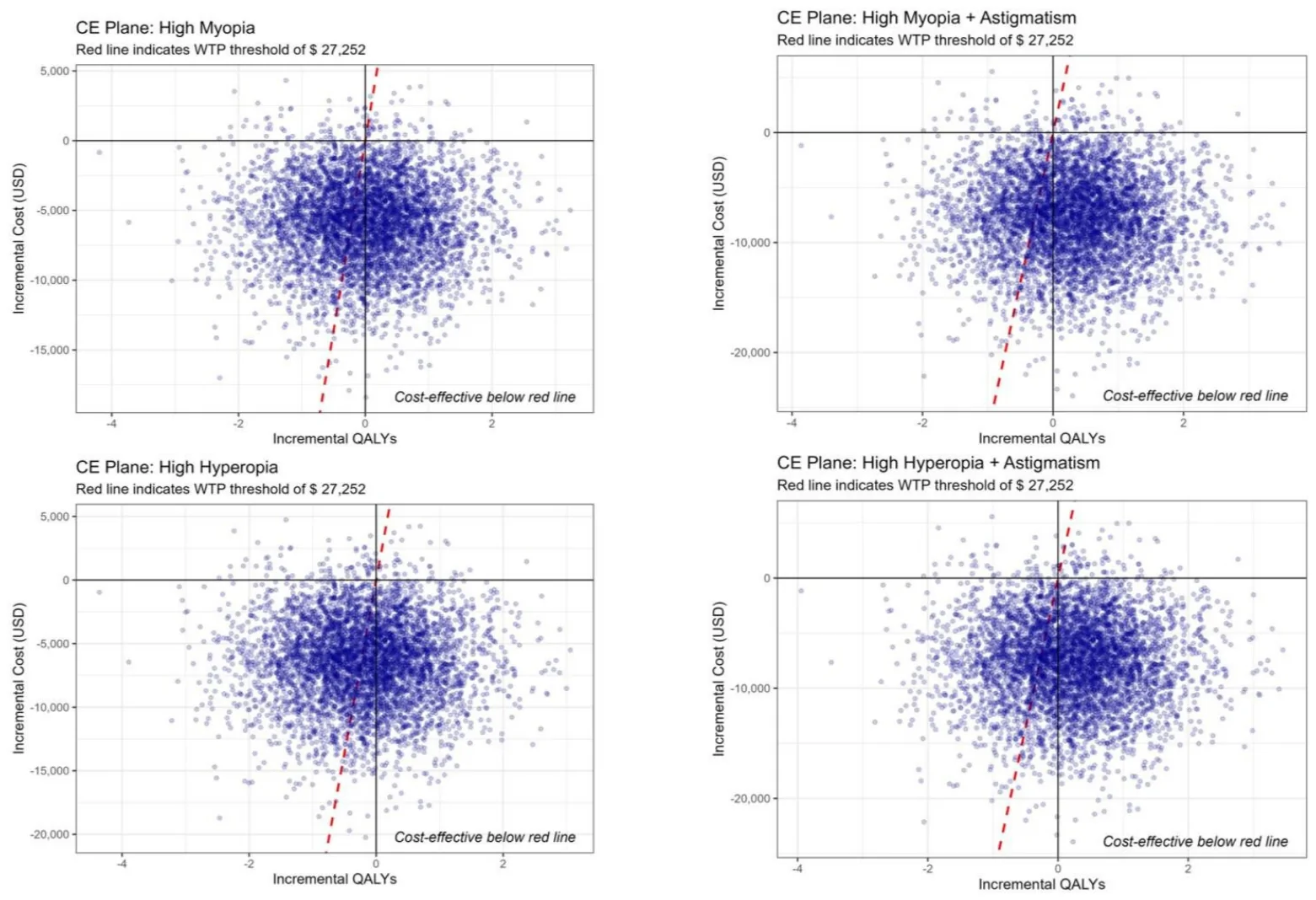
Cost-effectiveness planes of all studied subgroups.

Across all subgroups, the majority of simulations were located in the southeast quadrant (less costly, more effective), confirming a high probability of dominance. Dispersion was modest and primarily reflected uncertainty in utility parameters (Figure 1).

Even among simulations crossing into the northeast quadrant (more costly, more effective), the incremental QALY gains were sufficient to maintain a positive net benefit at conventional WTP thresholds.

### 3.8. Cost-Effective Acceptability Curve (CEAC)

**Figure 2:**
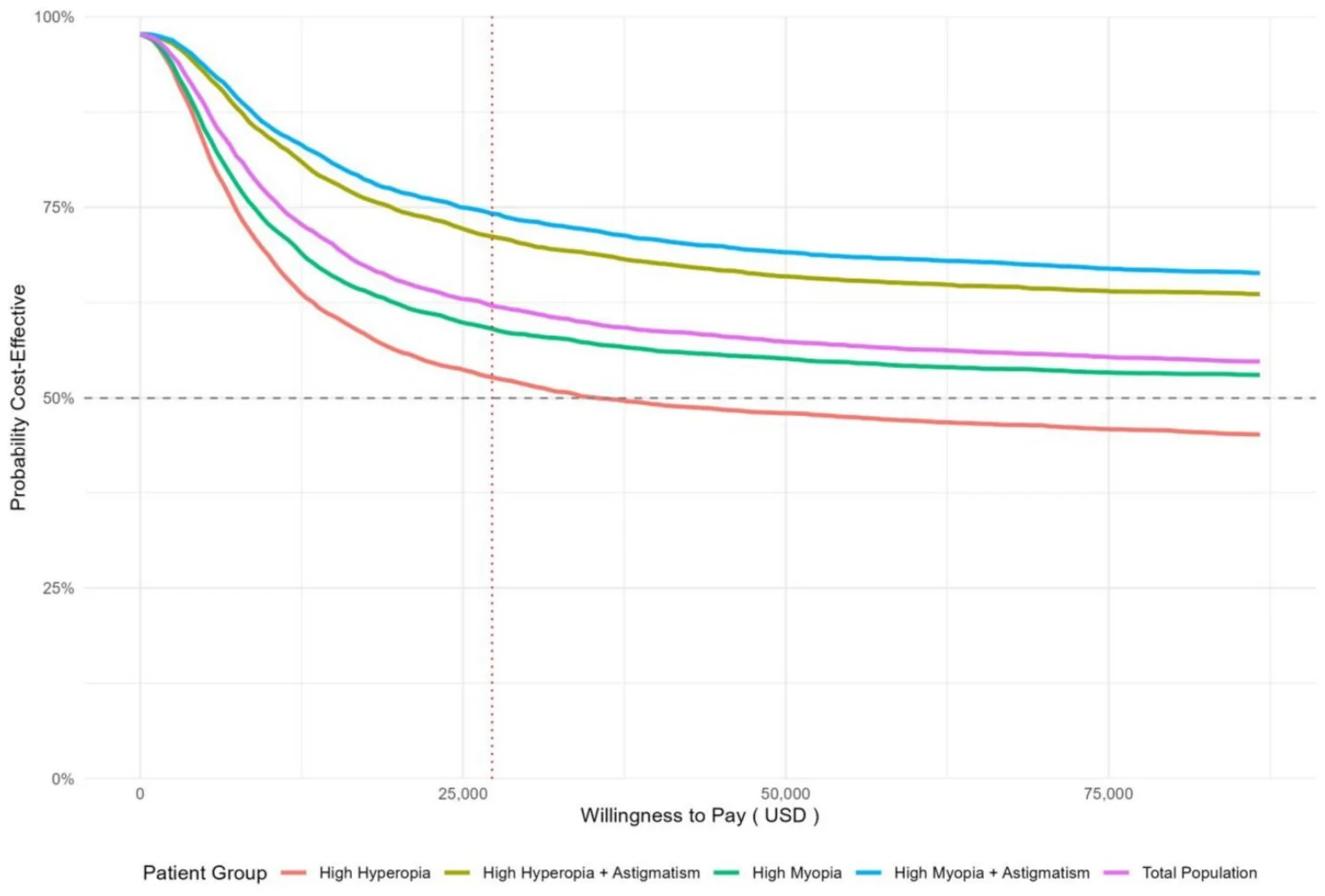
Cost-effective acceptability curve (USD), reflecting the probability of cost-effectiveness across varying willingness-to-pay thresholds.

At baseline analysis, all subgroups reflected a higher probability of cost-effectiveness above 50%, confirming the CE planes. At a higher WTP threshold (i.e., USD 35,000), the probability of ICL being cost-effective in the high-hyperopia subgroup fell below 50%. Nevertheless, in the weighted total population, ICL had almost a 60% probability of being cost-effective at the highest WTP threshold modelled (Figure 2).

### 3.9. Cost-Effectiveness Acceptability Frontier (Population Level)

**Figure 3:**
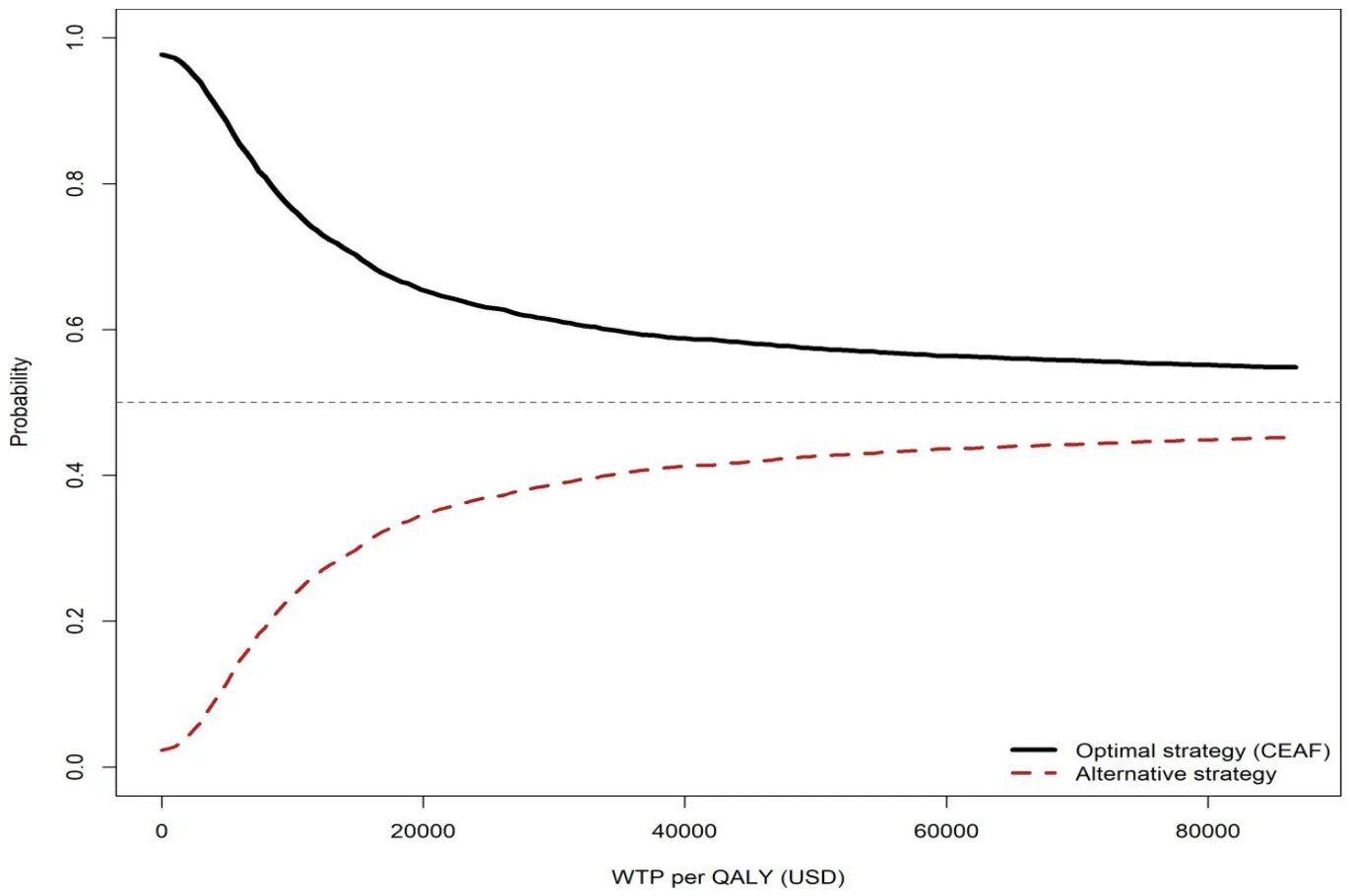
Cost-effective acceptability frontier (population-weighted, USD).

At the population-weighted level, ICL had approximately 97% probability of being optimal at very low WTP thresholds (<USD 5000 per QALY). As the WTP threshold increased to USD 20,000 and to 85,000 per QALY, the probability of ICL being optimal remained above 60% and 55%, respectively (Figure 3).

## 4. Discussion

This 25-year societal-perspective evaluation demonstrates that ICL implantation is more cost-effective than CL correction across four refractive subgroups. In all deterministic base-case analyses, ICL generated greater QALYs while reducing costs over the 25-year horizon period, with per-patient savings ranging from approximately USD 1758 (NOK 17,740) to USD 2277 (NOK 22,980) and QALY gains of 0.05–0.46. These findings were supported by probabilistic sensitivity analysis.

### 4.1. Principal Findings and Clinical Context

This long-term Markov model comparing ICL implantation with contact lens correction suggests that ICLs may be cost-effective or even dominant over a 25-year horizon from a societal perspective in Norway. ICL implantation offers substantial clinical benefits—including marked improvements in uncorrected visual acuity and reduced dependence on external optical aids—which translate into QALY gains compared with continuing contact lens use.

ICL surgery provides predictable and sustained refractive correction in appropriately selected patients [14,15,16,17,39,40,41]. These clinical findings are consistent with the model assumptions that improved visual functioning and reduced dependence on external correction generate utility gains.

### 4.2. Quality of Life and Patient-Reported Outcomes

Beyond objective refractive outcomes, ICL has been associated with better vision-related quality of life and patient satisfaction than contact lens wear [8,14]. Reduced daily burdens associated with contact lens discomfort, maintenance, and spectacle dependence provide plausible mechanisms for these benefits.

Importantly, these quality-of-life improvements carry economic significance. By enhancing functional vision and reducing dependency on contact lenses—which incur recurring costs for lenses, care solutions, replacements, and optometric visits—ICLs may generate long-term cost offsets. However, formal cost-utility analyses specifically evaluating phakic ICLs are rare. A provincial health technology assessment did not identify robust economic evaluations of posterior chamber phakic intraocular lenses for refractive error correction (Medical Advisory Secretariat, 2009). This highlights a gap in the literature and underscores the value of the present modelling effort.

### 4.3. Safety Considerations

The long-term safety profile of ICL is a central determinant of value. Studies of modern central-port lenses generally report high efficacy and low rates of serious events, although endothelial cell loss, cataract formation, elevated intraocular pressure, lens rotation, and explantation require ongoing surveillance [14,15,16,17,39,40,41,42].

Complications can reduce quality of life and economic value through additional treatment and monitoring [16,42]. The model therefore includes cataract, temporary complications, explantation, microbial keratitis, and permanent visual loss and tests uncertainty in the corresponding event rates.

### 4.4. Economic Implications and Societal Perspective

From a societal perspective, refractive error can generate direct correction costs and productivity losses [3]. The potential for ICL to offset these costs arises from reduced recurring expenses for contact lenses, care solutions, spectacles, and optometric visits. Evidence from refractive-surgery costing studies supports this mechanism, although direct cost-utility evidence for ICL remains limited [9,10].

In Norway—with an aging population and high prevalence of myopia—interventions that reduce long-term dependence on corrective devices could yield meaningful economic value. A model that captures differences in costs (surgery versus recurring contact lens expenses) and QALY gains can inform reimbursement and clinical guidance decisions. Further country-specific data on costs, patient preferences, and health utility values would strengthen policy relevance.

Perspective affects decision relevance. A payer analysis excludes travel and productivity effects and therefore narrows the estimated cost difference. Because direct medical costs are health-service costs rather than societal costs, the model reports direct medical, direct non-medical, and productivity components separately. The societal base case remains appropriate for the study objective, whereas the qualitative payer interpretation is presented for reimbursement relevance.

The deterministic finding of lower cost and greater effectiveness is directionally consistent with the recurring-cost offsets reported for refractive surgery [10] and with the quality-of-life advantage reported for ICL over contact lenses [8]. However, those studies were conducted outside Norway and used different designs. The high-hyperopia PSA result further shows that small utility differences can alter the decision, supporting cautious interpretation and the need for prospective Norwegian data.

The 25-year horizon was chosen to represent working-age adulthood and should not be interpreted as a lifetime horizon. Stopping at age 50 avoids uncertain extrapolation but omits later cataract, retinal disease, other age-related ophthalmic conditions, ICL explantation, and continuing spectacle or CL costs. Extending the horizon could increase accumulated device-cost offsets while increasing age-related and ICL-related events; the net direction is therefore uncertain without explicit modelling. Results should be interpreted as 25-year projections.

Over the modelled 25-year horizon, ICL may provide economic and quality-of-life advantages over continued CL-based correction with backup spectacles in appropriately selected Norwegian adults. The declining probability with increasing WTP reflects the fact that although the deterministic base case favoured ICL in all four refractive subgroups, probabilistic confidence varied and was limited for high hyperopia. The result depends particularly on utility estimates, CL discontinuation, the valuation of productivity effects, and the treatment of cataract risk. Decision makers should therefore consider both societal and payer perspectives and should not interpret these projections as evidence over a 25-year period. Transparent reporting of transition matrices, model inputs, validation checks, and scenario analyses is cost-saving, as increasing WTP may reduce the relative impact of uncertainty in incremental QALY on the relative net benefit margin. Importantly, across all Norwegian-relevant thresholds (NOK 275,000–825,000 per QALY), ICL remained the strategy with the highest expected net benefit. All of these are essential before the model is used to support reimbursement or policy. Prospective Norwegian data on utilities, resource use, complications, and long-term outcomes would materially strengthen future estimates.

### 4.5. Strengths and Limitations

Key strengths of this analysis include the long-term horizon and the use of a Markov model to account for transition probabilities across health states that reflect real-world trajectories. By incorporating societal costs and utility gains, the model aligns with guidelines for health technology assessment in many jurisdictions.

However, several limitations must be acknowledged. The absence of prior cost-utility analyses for ICLs necessitated reliance on model assumptions and analogues from other refractive procedures (Medical Advisory Secretariat, 2009). Quality-of-life gains attributable to refractive independence may vary across populations and settings, affecting QALY estimates. Patient heterogeneity (age, baseline refractive error, lifestyle) can influence both utilities and cost outcomes. Although rare, surgical risks were modelled using simplifying assumptions. Future work could incorporate more granular data on adverse events and their economic impacts. Results derived from a Norwegian context may not directly extrapolate to other health systems with different cost structures and patient preferences.

### 4.6. Future Research

To address evidence gaps, future prospective studies should measure health utilities and societal costs specific to ICL implantation compared with long-term contact lens use. Randomized controlled or pragmatic observational studies that collect QALY data (e.g., via EQ-5D or similar instruments) alongside resource use would greatly strengthen economic evaluations. Furthermore, evaluations across different refractive error strata (e.g., high myopia) could elucidate subgroup-specific value profiles.

Finally, real-world cost data for contact lens maintenance, surgical complications, and long-term follow-up care would enhance model precision and transferability across health systems.

## 5. Conclusions

This modelling study indicates that ICLs may offer long-term economic and quality-of-life advantages over continued contact lens correction when evaluated from a societal perspective. Although direct cost-utility evidence remains sparse in the literature, the clinical benefits and potential cost offsets support consideration of ICLs as a high-value option for appropriately selected patients. Continued research is needed to further substantiate these findings and guide evidence-based decision-making in refractive care.

## Figures and Tables

**Table 1:** Characteristics of the simulated model cohort.

Characteristic	Base-Case Specification
Population	Norwegian adults eligible for ICL or continued CL-based correction
Age at model entry	25 years (eligible range 20–30 years)
Time horizon	25 annual cycles; ages 25–50 years
Subgroups	High myopia; high myopia with astigmatism; high hyperopia; high hyperopia with astigmatism
Sex and other demographics	Not stratified; no individual participants were enrolled
Perspective	Societal (direct medical, direct non-medical, and productivity costs)
Comparator	Continued contact-lens correction with backup spectacles

**Table 2:** Cost categories for the ICL vs. CL Markov Model.

Cost Category	Parameter	Base Cost NOK	Base Cost USD	Frequency
Direct Medical	ICL surgery cost	60,000.00	5946.00	one-time—total cost for both eyes
	ICL follow-up year 1	1410.56	139.79	one-time
	ICL follow-up later	2194.42	217.47	per year
	ICL explant cost	8882.16	880.22	per event
	Cataract surgery cost	12,330.53	1221.96	per event
	CL initial fit	1200.00	118.92	one-time
	CL annual cost	6000.00	594.60	per year (Annual cost for contact lenses, solutions, and routine visits)
	CL MK treatment	73,356.19	7269.60	per event
	CL Post MK visual loss cost	360,000.00	35,676.00	per year
	Glasses cost	4000.00	396.40	every 2 years
	Outpatients visit fee	821.00	81.36	per visit
	Back-up glasses cost	4500.00	445.95	every 3 years
	Travel per visit	1728.00	171.24	per visit
Indirect Medical	Productivity loss surgery	6112.50	605.75	per surgery
	Productivity loss follow-up	592.50	58.72	per visit
	Societal annual salary	720,000.00	71,352.00	Per year (0.45 unemployment productivity loss visual impairment)

ICL: Implantable Collamer Lens, CL: Contact Lens, MK: Microbial Keratitis.

**Table 3:** Base-case cost-effectiveness results for implantable collamer lens implantation versus contact lens-based correction over the 25-year model horizon.

Subgroup	CL Cost USD	ICL Cost USD	CL QALY	ICL QALY	dQAL Y	dCost USD	INMB USD	ICER USD
High Myopia	12,770.39	11,012.62	15.598	15.815	0.217	−1757.76	7662.22	ICL Dominates
High Myopia + Astigmatism	16,669.83	14,396.53	15.51	15.97	0.464	−2273.30	14,921.11	ICL Dominates
High Hyperopia	14,066.70	12,136.63	15.60	15.65	0.049	−1930.07	3264.28	ICL Dominates
High Hyperopia + Astigmatism	16,647.06	14,370.00	15.51	15.89	0.385	−2277.06	12,762.07	ICL Dominates

**Table 4:** Norway priority-setting threshold sensitivity.

Subgroup	Low Severity 250k	Base Case 275k	Medium Severity 600k	High Severity 825k
High Myopia	60%	59.10%	54.20%	53.20%
High Myopia + Astigmatism	75%	74.20%	68.20%	66.60%
High Hyperopia	53.80%	52.70%	47.10%	45.50%
High Hyperopia + Astigmatism	72.30%	71.20%	65.10%	63.90%

## Data Availability

The original contributions presented in this study are included in the article/Supplementary Materials. Further inquiries can be directed to the corresponding author.

## Supplementary Materials

The following supporting information can be downloaded at https://www.mdpi.com/article/10.3390/healthcare14193220/s1: Table S1, annual transition rules by strategy; Table S2, base-case utilities, event probabilities, costs, uncertainty distributions, bounds, and sources. Supplementary Figure S1 contains Markov traces, Supplementary Figure S2 contains model inputs, and Supplementary Figure S3 contains deterministic sensitivity analyses.
