# Supplementary material for: Cost-Effectiveness Analysis of Implantable Collamer Lens (ICL) Surgery Versus Contact Lens-Based Correction with Backup Spectacles in Young Adults—A Norwegian Societal Perspective
Source: Healthcare (Basel). 2026 Sep 30;14(19):3220. doi: 10.3390/healthcare14193220 (PMC13664969; doi:10.3390/healthcare14193220)
Supplement: Supplementary file 1 [file healthcare-14-03220-s001.zip › healthcare-4453534-supplementary.pdf]

Supplementary Figures and Tables

Supplementary Figure S1. Markov Traces in the study conditions.

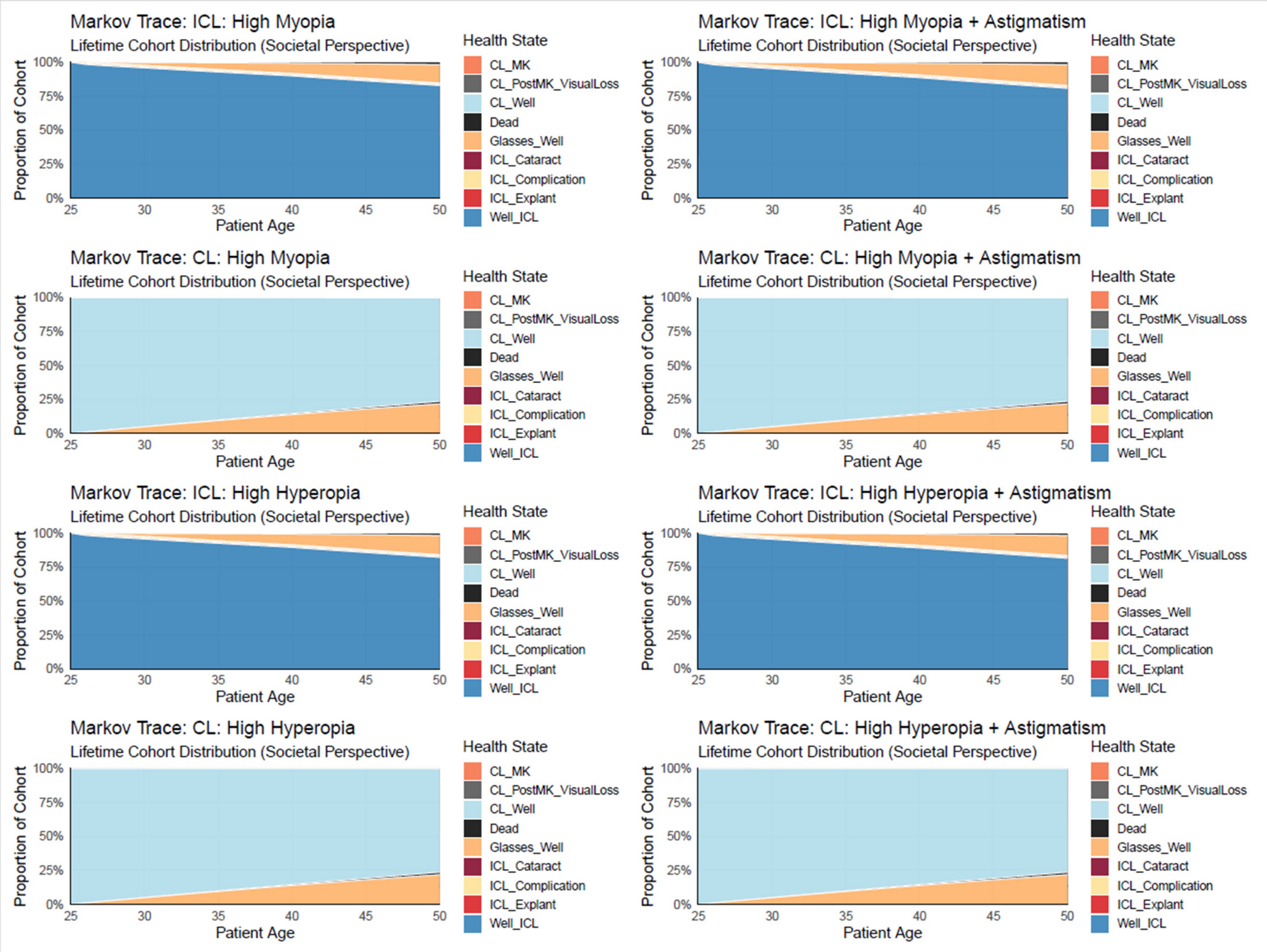

Supplementary Figure S2. Expected Value of Perfect Information (EVPI), The expected value of perfect information (EVPI), estimated for a representative patient using population-weighted model inputs, increased with increasing willingness-to-pay (WTP) thresholds. EVPI was close to zero at low WTP values and increased steadily, reaching approximately 540,000 NOK at a WTP threshold of 875,000 NOK/QALY. This indicates that the value of eliminating parameter uncertainty increases as the WTP threshold increases.

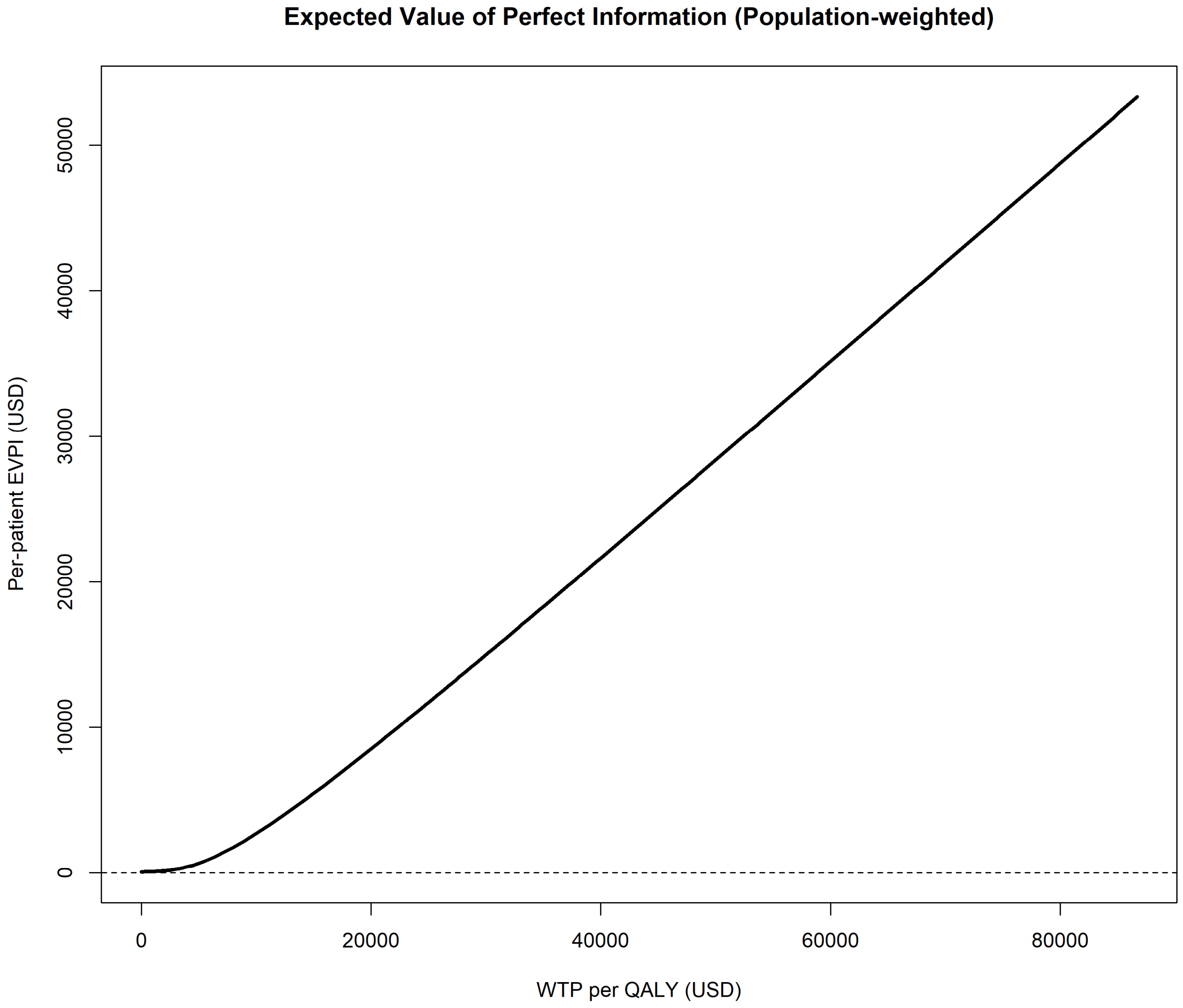

Supplementary Figure S3. Deterministic sensitivity analyses for the study conditions.

A

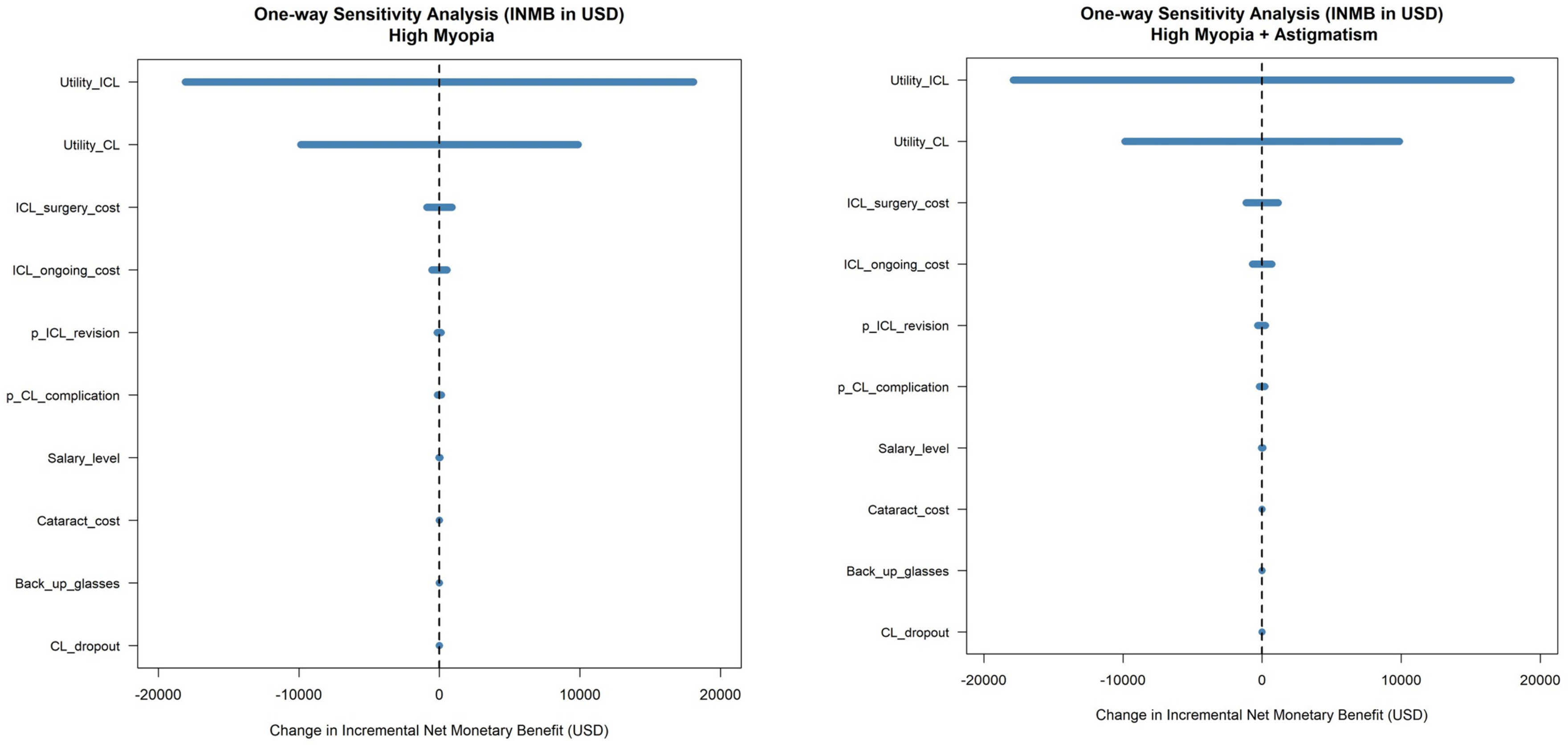

B

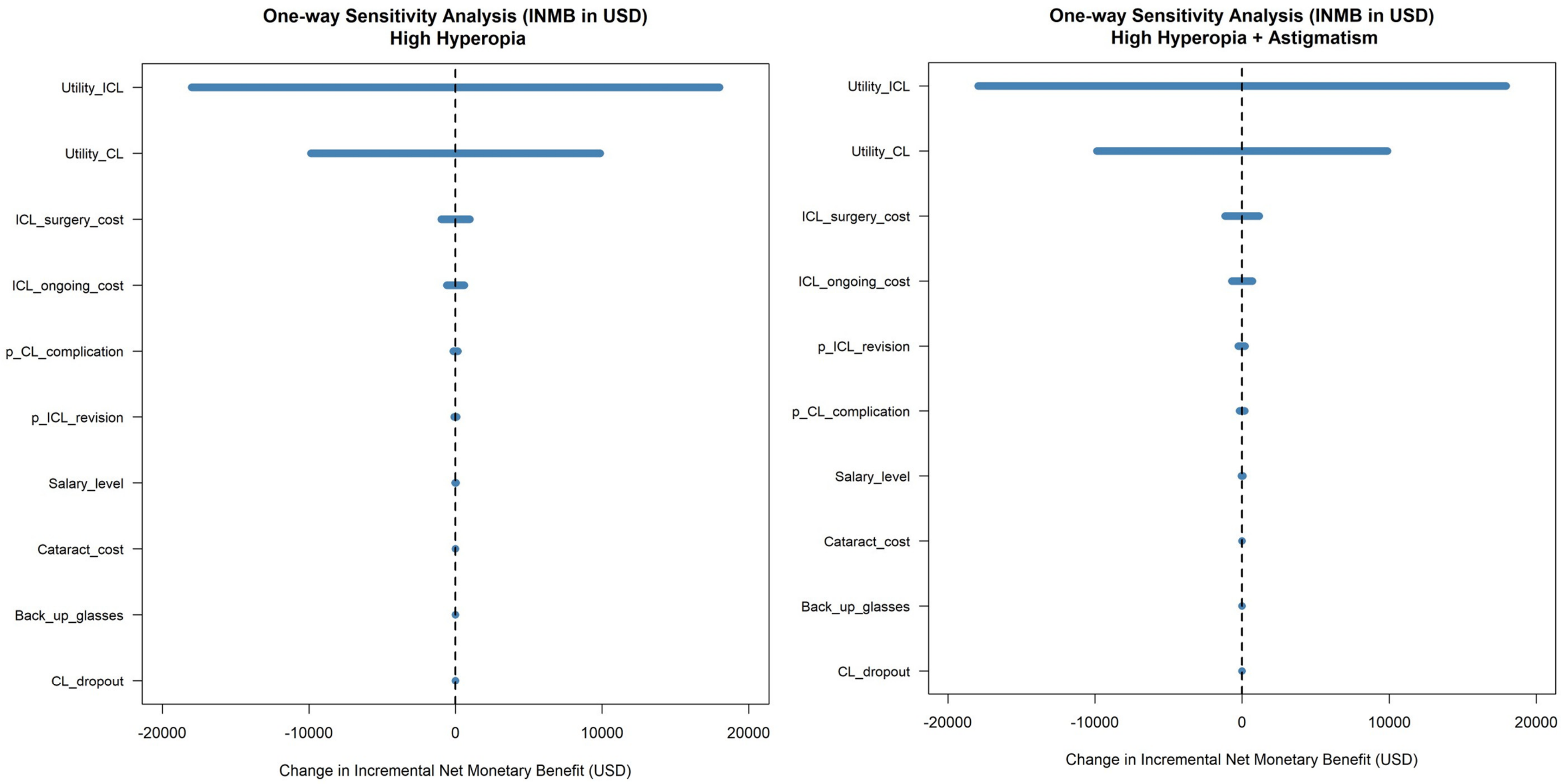
One-way Sensitivity Analysis (INMB in USD)
High Hyperopia
Utility_ICL
Utility_CL
ICL_surgery_cost
ICL_ongoing_cost
p_CL_complication
p_ICL_revision
Salary_level
Cataract_cost
Back_up_glasses
CL_dropout
-20000
-10000
0
10000
20000
Change in Incremental Net Monetary Benefit (USD)
One-way Sensitivity Analysis (INMB in USD)
High Hyperopia + Astigmatism
Utility_ICL
Utility_CL
ICL_surgery_cost
ICL_ongoing_cost
p_ICL_revision
p_CL_complication
Salary_level
Cataract_cost
Back_up_glasses
CL_dropout
-20000
-10000
0
10000
20000
Change in Incremental Net Monetary Benefit (USD)

Supplementary Table S1. Transition age matrix.

**Year 1_Age 25**									
	Well_ICL	ICL_Complication	ICL_Explant	ICL_Cataract	CL_Well	CL_MK	CL_PostMK_Visual Loss	Glasses_Well	Dead
Well_ICL	0.98403867	0.01	0.005	0.0005	0	0	0	0	0.000461
ICL_Complication	0.8	0.16953867	0.02	0.01	0	0	0	0	0.000461
ICL_Explant	0	0	0.01953867	0	0	0	0	0.98	0.000461
ICL_Cataract	0	0	0.2	0.199539	0	0	0	0.6	0.000461
CL_Well	0	0	0	0	0.989039	0.0005	0	0.01	0.000461
CL_MK	0	0	0	0	0.89	0.009539	0.1	0	0.000461
CL_PostMK_Visual Loss	0	0	0	0	0	0	0.999539	0	0.000461
Glasses_Well	0.01	0	0	0	0	0	0	0.989539	0.000461
Dead	0	0	0	0	0	0	0	0	1
**Year 2_Age 26**									
	Well_ICL	ICL_Complication	ICL_Explant	ICL_Cataract	CL_Well	CL_MK	CL_PostMK_Visual Loss	Glasses_Well	Dead
Well_ICL	0.98407433	0.01	0.005	0.0005	0	0	0	0	0.000426
ICL_Complication	0.8	0.16957433	0.02	0.01	0	0	0	0	0.000426
ICL_Explant	0	0	0.01957433	0	0	0	0	0.98	0.000426

ICL_Cataract	0	0	0.2	0.199574	0	0	0	0.6	0.000426
CL_Well	0	0	0	0	0.989074	0.0005	0	0.01	0.000426
CL_MK	0	0	0	0	0.89	0.009574	0.1	0	0.000426
CL_PostMK_Visual Loss	0	0	0	0	0	0	0.999574	0	0.000426
Glasses_Well	0.01	0	0	0	0	0	0	0.989574	0.000426
Dead	0	0	0	0	0	0	0	0	1
**Year 3_Age 27**									
	Well_ICL	ICL_Complication	ICL_Explant	ICL_Cataract	CL_Well	CL_MK	CL_PostMK_Visual Loss	Glasses_Well	Dead
Well_ICL	0.984073	0.01	0.005	0.0005	0	0	0	0	0.000427
ICL_Complication	0.8	0.169573	0.02	0.01	0	0	0	0	0.000427
ICL_Explant	0	0	0.019573	0	0	0	0	0.98	0.000427
ICL_Cataract	0	0	0.2	0.199573	0	0	0	0.6	0.000427
CL_Well	0	0	0	0	0.989073	0.0005	0	0.01	0.000427
CL_MK	0	0	0	0	0.89	0.009573	0.1	0	0.000427
CL_PostMK_Visual Loss	0	0	0	0	0	0	0.999573	0	0.000427
Glasses_Well	0.01	0	0	0	0	0	0	0.989573	0.000427

Dead	0	0	0	0	0	0	0	0	1
**Year 4_Age 28**									
	Well_ICL	ICL_Complication	ICL_Explant	ICL_Cataract	CL_Well	CL_MK	CL_PostMK_Visual Loss	Glasses_Well	Dead
Well_ICL	0.98398833	0.01	0.005	0.0005	0	0	0	0	0.000512
ICL_Complication	0.8	0.16948833	0.02	0.01	0	0	0	0	0.000512
ICL_Explant	0	0	0.01948833	0	0	0	0	0.98	0.000512
ICL_Cataract	0	0	0.2	0.199488	0	0	0	0.6	0.000512
CL_Well	0	0	0	0	0.988988	0.0005	0	0.01	0.000512
CL_MK	0	0	0	0	0.89	0.009488	0.1	0	0.000512
CL_PostMK_Visual Loss	0	0	0	0	0	0	0.999488	0	0.000512
Glasses_Well	0.01	0	0	0	0	0	0	0.989488	0.000512
Dead	0	0	0	0	0	0	0	0	1
**Year 5_Age 29**									
	Well_ICL	ICL_Complication	ICL_Explant	ICL_Cataract	CL_Well	CL_MK	CL_PostMK_Visual Loss	Glasses_Well	Dead
Well_ICL	0.983989	0.01	0.005	0.0005	0	0	0	0	0.000511
ICL_Complication	0.8	0.169489	0.02	0.01	0	0	0	0	0.000511

ICL_Explant	0	0	0.019489	0	0	0	0	0.98	0.000511
ICL_Cataract	0	0	0.2	0.199489	0	0	0	0.6	0.000511
CL_Well	0	0	0	0	0.988989	0.0005	0	0.01	0.000511
CL_MK	0	0	0	0	0.89	0.009489	0.1	0	0.000511
CL_PostMK_Visual Loss	0	0	0	0	0	0	0.999489	0	0.000511
Glasses_Well	0.01	0	0	0	0	0	0	0.989489	0.000511
Dead	0	0	0	0	0	0	0	0	1
**Year 6_Age 30**									
	Well_ICL	ICL_Complication	ICL_Explant	ICL_Cataract	CL_Well	CL_MK	CL_PostMK_Visual Loss	Glasses_Well	Dead
Well_ICL	0.983688	0.01	0.005	0.001	0	0	0	0	0.000312
ICL_Complication	0.8	0.169688	0.02	0.01	0	0	0	0	0.000312
ICL_Explant	0	0	0.019688	0	0	0	0	0.98	0.000312
ICL_Cataract	0	0	0.2	0.199688	0	0	0	0.6	0.000312
CL_Well	0	0	0	0	0.989188	0.0005	0	0.01	0.000312
CL_MK	0	0	0	0	0.89	0.009688	0.1	0	0.000312
CL_PostMK_Visual Loss	0	0	0	0	0	0	0.999688	0	0.000312

Glasses_Well	0.01	0	0	0	0	0	0	0.989688	0.000312
Dead	0	0	0	0	0	0	0	0	1
**Year 7_Age 31**									
	Well_ICL	ICL_Complication	ICL_Explant	ICL_Cataract	CL_Well	CL_MK	CL_PostMK_Visual Loss	Glasses_Well	Dead
Well_ICL	0.983556	0.01	0.005	0.001	0	0	0	0	0.000444
ICL_Complication	0.8	0.169556	0.02	0.01	0	0	0	0	0.000444
ICL_Explant	0	0	0.019556	0	0	0	0	0.98	0.000444
ICL_Cataract	0	0	0.2	0.199556	0	0	0	0.6	0.000444
CL_Well	0	0	0	0	0.989056	0.0005	0	0.01	0.000444
CL_MK	0	0	0	0	0.89	0.009556	0.1	0	0.000444
CL_PostMK_Visual Loss	0	0	0	0	0	0	0.999556	0	0.000444
Glasses_Well	0.01	0	0	0	0	0	0	0.989556	0.000444
Dead	0	0	0	0	0	0	0	0	1
**Year 8_Age 32**									
	Well_ICL	ICL_Complication	ICL_Explant	ICL_Cataract	CL_Well	CL_MK	CL_PostMK_Visual Loss	Glasses_Well	Dead
Well_ICL	0.98350233	0.01	0.005	0.001	0	0	0	0	0.000498

ICL_Complication	0.8	0.16950233	0.02	0.01	0	0	0	0	0.000498
ICL_Explant	0	0	0.01950233	0	0	0	0	0.98	0.000498
ICL_Cataract	0	0	0.2	0.199502	0	0	0	0.6	0.000498
CL_Well	0	0	0	0	0.989002	0.0005	0	0.01	0.000498
CL_MK	0	0	0	0	0.89	0.009502	0.1	0	0.000498
CL_PostMK_Visual Loss	0	0	0	0	0	0	0.999502	0	0.000498
Glasses_Well	0.01	0	0	0	0	0	0	0.989502	0.000498
Dead	0	0	0	0	0	0	0	0	1
**Year 9_Age 33**									
	Well_ICL	ICL_Complication	ICL_Explant	ICL_Cataract	CL_Well	CL_MK	CL_PostMK_Visual Loss	Glasses_Well	Dead
Well_ICL	0.983546	0.01	0.005	0.001	0	0	0	0	0.000454
ICL_Complication	0.8	0.169546	0.02	0.01	0	0	0	0	0.000454
ICL_Explant	0	0	0.019546	0	0	0	0	0.98	0.000454
ICL_Cataract	0	0	0.2	0.199546	0	0	0	0.6	0.000454
CL_Well	0	0	0	0	0.989046	0.0005	0	0.01	0.000454
CL_MK	0	0	0	0	0.89	0.009546	0.1	0	0.000454

CL_PostMK_Visual Loss	0	0	0	0	0	0	0.999546	0	0.0004 54
Glasses_Well	0.01	0	0	0	0	0	0	0.989546	0.0004 54
Dead	0	0	0	0	0	0	0	0	1
**Year 10_Age 34**									
	Well_IC L	ICL_Complica tion	ICL_Expl ant	ICL_Catar act	CL_W ell	CL_M K	CL_PostMK_Visual Loss	Glasses_ Well	Dead
Well_ICL	0.983361 33	0.01	0.005	0.001	0	0	0	0	0.0006 39
ICL_Complication	0.8	0.16936133	0.02	0.01	0	0	0	0	0.0006 39
ICL_Explant	0	0	0.0193613 3	0	0	0	0	0.98	0.0006 39
ICL_Cataract	0	0	0.2	0.199361	0	0	0	0.6	0.0006 39
CL_Well	0	0	0	0	0.9888 61	0.0005	0	0.01	0.0006 39
CL_MK	0	0	0	0	0.89	0.0093 61	0.1	0	0.0006 39
CL_PostMK_Visual Loss	0	0	0	0	0	0	0.999361	0	0.0006 39
Glasses_Well	0.01	0	0	0	0	0	0	0.989361	0.0006 39
Dead	0	0	0	0	0	0	0	0	1
Year 11_Age 35									
	Well_IC L	ICL_Complica tion	ICL_Expl ant	ICL_Catar act	CL_W ell	CL_M K	CL_PostMK_Visual Loss	Glasses_ Well	Dead

Well_ICL	0.983503	0.01	0.005	0.001	0	0	0	0	0.000497
ICL_Complication	0.8	0.169503	0.02	0.01	0	0	0	0	0.000497
ICL_Explant	0	0	0.019503	0	0	0	0	0.98	0.000497
ICL_Cataract	0	0	0.2	0.199503	0	0	0	0.6	0.000497
CL_Well	0	0	0	0	0.989003	0.0005	0	0.01	0.000497
CL_MK	0	0	0	0	0.89	0.009503	0.1	0	0.000497
CL_PostMK_Visual Loss	0	0	0	0	0	0	0.999503	0	0.000497
Glasses_Well	0.01	0	0	0	0	0	0	0.989503	0.000497
Dead	0	0	0	0	0	0	0	0	1
**Year 12_Age 36**									
	Well_ICL	ICL_Complication	ICL_Explant	ICL_Cataract	CL_Well	CL_MK	CL_PostMK_Visual Loss	Glasses_Well	Dead
Well_ICL	0.98351967	0.01	0.005	0.001	0	0	0	0	0.00048
ICL_Complication	0.8	0.16951967	0.02	0.01	0	0	0	0	0.00048
ICL_Explant	0	0	0.01951967	0	0	0	0	0.98	0.00048
ICL_Cataract	0	0	0.2	0.19952	0	0	0	0.6	0.00048
CL_Well	0	0	0	0	0.98902	0.0005	0	0.01	0.00048

CL_MK	0	0	0	0	0.89	0.00952	0.1	0	0.00048
CL_PostMK_Visual Loss	0	0	0	0	0	0	0.99952	0	0.00048
Glasses_Well	0.01	0	0	0	0	0	0	0.98952	0.00048
Dead	0	0	0	0	0	0	0	0	1
**Year 13_Age 37**									
	Well_ICL	ICL_Complication	ICL_Explant	ICL_Cataract	CL_Well	CL_MK	CL_PostMK_Visual Loss	Glasses_Well	Dead
Well_ICL	0.98341833	0.01	0.005	0.001	0	0	0	0	0.000582
ICL_Complication	0.8	0.16941833	0.02	0.01	0	0	0	0	0.000582
ICL_Explant	0	0	0.01941833	0	0	0	0	0.98	0.000582
ICL_Cataract	0	0	0.2	0.199418	0	0	0	0.6	0.000582
CL_Well	0	0	0	0	0.988918	0.0005	0	0.01	0.000582
CL_MK	0	0	0	0	0.89	0.009418	0.1	0	0.000582
CL_PostMK_Visual Loss	0	0	0	0	0	0	0.999418	0	0.000582
Glasses_Well	0.01	0	0	0	0	0	0	0.989418	0.000582
Dead	0	0	0	0	0	0	0	0	1
**Year 14_Age 38**									

	Well_ICL	ICL_Complication	ICL_Explant	ICL_Cataract	CL_Well	CL_MK	CL_PostMK_Visual Loss	Glasses_Well	Dead
Well_ICL	0.98339867	0.01	0.005	0.001	0	0	0	0	0.000601
ICL_Complication	0.8	0.16939867	0.02	0.01	0	0	0	0	0.000601
ICL_Explant	0	0	0.01939867	0	0	0	0	0.98	0.000601
ICL_Cataract	0	0	0.2	0.199399	0	0	0	0.6	0.000601
CL_Well	0	0	0	0	0.988899	0.0005	0	0.01	0.000601
CL_MK	0	0	0	0	0.89	0.009399	0.1	0	0.000601
CL_PostMK_Visual Loss	0	0	0	0	0	0	0.999399	0	0.000601
Glasses_Well	0.01	0	0	0	0	0	0	0.989399	0.000601
Dead	0	0	0	0	0	0	0	0	1
**Year 15_Age 39**									
	Well_ICL	ICL_Complication	ICL_Explant	ICL_Cataract	CL_Well	CL_MK	CL_PostMK_Visual Loss	Glasses_Well	Dead
Well_ICL	0.98305633	0.01	0.005	0.001	0	0	0	0	0.000944
ICL_Complication	0.8	0.16905633	0.02	0.01	0	0	0	0	0.000944
ICL_Explant	0	0	0.01905633	0	0	0	0	0.98	0.000944

ICL_Cataract	0	0	0.2	0.199056	0	0	0	0.6	0.000944
CL_Well	0	0	0	0	0.988556	0.0005	0	0.01	0.000944
CL_MK	0	0	0	0	0.89	0.009056	0.1	0	0.000944
CL_PostMK_Visual Loss	0	0	0	0	0	0	0.999056	0	0.000944
Glasses_Well	0.01	0	0	0	0	0	0	0.989056	0.000944
Dead	0	0	0	0	0	0	0	0	1
**Year 16_Age 40**									
	Well_ICL	ICL_Complication	ICL_Explant	ICL_Cataract	CL_Well	CL_MK	CL_PostMK_Visual Loss	Glasses_Well	Dead
Well_ICL	0.981029	0.01	0.005	0.003	0	0	0	0	0.000971
ICL_Complication	0.8	0.169029	0.02	0.01	0	0	0	0	0.000971
ICL_Explant	0	0	0.019029	0	0	0	0	0.98	0.000971
ICL_Cataract	0	0	0.2	0.199029	0	0	0	0.6	0.000971
CL_Well	0	0	0	0	0.988529	0.0005	0	0.01	0.000971
CL_MK	0	0	0	0	0.89	0.009029	0.1	0	0.000971
CL_PostMK_Visual Loss	0	0	0	0	0	0	0.999029	0	0.000971
Glasses_Well	0.01	0	0	0	0	0	0	0.989029	0.000971

Dead	0	0	0	0	0	0	0	0	1
**Year 17_Age 41**									
	Well_ICL	ICL_Complication	ICL_Explant	ICL_Cataract	CL_Well	CL_MK	CL_PostMK_Visual Loss	Glasses_Well	Dead
Well_ICL	0.98116667	0.01	0.005	0.003	0	0	0	0	0.000833
ICL_Complication	0.8	0.16916667	0.02	0.01	0	0	0	0	0.000833
ICL_Explant	0	0	0.01916667	0	0	0	0	0.98	0.000833
ICL_Cataract	0	0	0.2	0.199167	0	0	0	0.6	0.000833
CL_Well	0	0	0	0	0.988667	0.0005	0	0.01	0.000833
CL_MK	0	0	0	0	0.89	0.009167	0.1	0	0.000833
CL_PostMK_Visual Loss	0	0	0	0	0	0	0.999167	0	0.000833
Glasses_Well	0.01	0	0	0	0	0	0	0.989167	0.000833
Dead	0	0	0	0	0	0	0	0	1
**Year 18_Age 42**									
	Well_ICL	ICL_Complication	ICL_Explant	ICL_Cataract	CL_Well	CL_MK	CL_PostMK_Visual Loss	Glasses_Well	Dead
Well_ICL	0.98121	0.01	0.005	0.003	0	0	0	0	0.00079
ICL_Complication	0.8	0.16921	0.02	0.01	0	0	0	0	0.00079

ICL_Explant	0	0	0.01921	0	0	0	0	0.98	0.00079
ICL_Cataract	0	0	0.2	0.19921	0	0	0	0.6	0.00079
CL_Well	0	0	0	0	0.98871	0.0005	0	0.01	0.00079
CL_MK	0	0	0	0	0.89	0.00921	0.1	0	0.00079
CL_PostMK_Visual Loss	0	0	0	0	0	0	0.99921	0	0.00079
Glasses_Well	0.01	0	0	0	0	0	0	0.98921	0.00079
Dead	0	0	0	0	0	0	0	0	1
**Year 19_Age 43**									
	Well_ICL	ICL_Complication	ICL_Explant	ICL_Cataract	CL_Well	CL_MK	CL_PostMK_Visual Loss	Glasses_Well	Dead
Well_ICL	0.98109433	0.01	0.005	0.003	0	0	0	0	0.000906
ICL_Complication	0.8	0.16909433	0.02	0.01	0	0	0	0	0.000906
ICL_Explant	0	0	0.01909433	0	0	0	0	0.98	0.000906
ICL_Cataract	0	0	0.2	0.199094	0	0	0	0.6	0.000906
CL_Well	0	0	0	0	0.988594	0.0005	0	0.01	0.000906
CL_MK	0	0	0	0	0.89	0.009094	0.1	0	0.000906
CL_PostMK_Visual Loss	0	0	0	0	0	0	0.999094	0	0.000906

Glasses_Well	0.01	0	0	0	0	0	0	0.989094	0.000906
Dead	0	0	0	0	0	0	0	0	1
**Year 20_Age 44**									
	Well_ICL	ICL_Complication	ICL_Explant	ICL_Cataract	CL_Well	CL_MK	CL_PostMK_Visual Loss	Glasses_Well	Dead
Well_ICL	0.980968	0.01	0.005	0.003	0	0	0	0	0.001032
ICL_Complication	0.8	0.168968	0.02	0.01	0	0	0	0	0.001032
ICL_Explant	0	0	0.018968	0	0	0	0	0.98	0.001032
ICL_Cataract	0	0	0.2	0.198968	0	0	0	0.6	0.001032
CL_Well	0	0	0	0	0.988468	0.0005	0	0.01	0.001032
CL_MK	0	0	0	0	0.89	0.008968	0.1	0	0.001032
CL_PostMK_Visual Loss	0	0	0	0	0	0	0.998968	0	0.001032
Glasses_Well	0.01	0	0	0	0	0	0	0.988968	0.001032
Dead	0	0	0	0	0	0	0	0	1
**Year 21_Age 45**									
	Well_ICL	ICL_Complication	ICL_Explant	ICL_Cataract	CL_Well	CL_MK	CL_PostMK_Visual Loss	Glasses_Well	Dead
Well_ICL	0.98086833	0.01	0.005	0.003	0	0	0	0	0.001132

ICL_Complication	0.8	0.16886833	0.02	0.01	0	0	0	0	0.001132
ICL_Explant	0	0	0.01886833	0	0	0	0	0.98	0.001132
ICL_Cataract	0	0	0.2	0.198868	0	0	0	0.6	0.001132
CL_Well	0	0	0	0	0.988368	0.0005	0	0.01	0.001132
CL_MK	0	0	0	0	0.89	0.008868	0.1	0	0.001132
CL_PostMK_Visual Loss	0	0	0	0	0	0	0.998868	0	0.001132
Glasses_Well	0.01	0	0	0	0	0	0	0.988868	0.001132
Dead	0	0	0	0	0	0	0	0	1
**Year 22_Age 46**									
	Well_ICL	ICL_Complication	ICL_Explant	ICL_Cataract	CL_Well	CL_MK	CL_PostMK_Visual Loss	Glasses_Well	Dead
Well_ICL	0.98090533	0.01	0.005	0.003	0	0	0	0	0.001095
ICL_Complication	0.8	0.16890533	0.02	0.01	0	0	0	0	0.001095
ICL_Explant	0	0	0.01890533	0	0	0	0	0.98	0.001095
ICL_Cataract	0	0	0.2	0.198905	0	0	0	0.6	0.001095
CL_Well	0	0	0	0	0.988405	0.0005	0	0.01	0.001095
CL_MK	0	0	0	0	0.89	0.008905	0.1	0	0.001095

CL_PostMK_Visual Loss	0	0	0	0	0	0	0.998905	0	0.0010 95
Glasses_Well	0.01	0	0	0	0	0	0	0.988905	0.0010 95
Dead	0	0	0	0	0	0	0	0	1
**Year 23_Age 47**									
	Well_IC L	ICL_Complica tion	ICL_Expl ant	ICL_Catar act	CL_W ell	CL_M K	CL_PostMK_Visual Loss	Glasses_ Well	Dead
Well_ICL	0.980669 67	0.01	0.005	0.003	0	0	0	0	0.0013 3
ICL_Complication	0.8	0.16866967	0.02	0.01	0	0	0	0	0.0013 3
ICL_Explant	0	0	0.0186696 7	0	0	0	0	0.98	0.0013 3
ICL_Cataract	0	0	0.2	0.19867	0	0	0	0.6	0.0013 3
CL_Well	0	0	0	0	0.9881 7	0.0005	0	0.01	0.0013 3
CL_MK	0	0	0	0	0.89	0.0086 7	0.1	0	0.0013 3
CL_PostMK_Visual Loss	0	0	0	0	0	0	0.99867	0	0.0013 3
Glasses_Well	0.01	0	0	0	0	0	0	0.98867	0.0013 3
Dead	0	0	0	0	0	0	0	0	1
**Year 24_Age 48**									
	Well_IC L	ICL_Complica tion	ICL_Expl ant	ICL_Catar act	CL_W ell	CL_M K	CL_PostMK_Visual Loss	Glasses_ Well	Dead

Well_ICL	0.980787	0.01	0.005	0.003	0	0	0	0	0.001213
ICL_Complication	0.8	0.168787	0.02	0.01	0	0	0	0	0.001213
ICL_Explant	0	0	0.018787	0	0	0	0	0.98	0.001213
ICL_Cataract	0	0	0.2	0.198787	0	0	0	0.6	0.001213
CL_Well	0	0	0	0	0.988287	0.0005	0	0.01	0.001213
CL_MK	0	0	0	0	0.89	0.008787	0.1	0	0.001213
CL_PostMK_Visual Loss	0	0	0	0	0	0	0.998787	0	0.001213
Glasses_Well	0.01	0	0	0	0	0	0	0.988787	0.001213
Dead	0	0	0	0	0	0	0	0	1
**Year 25_Age 49**									
	Well_ICL	ICL_Complication	ICL_Explant	ICL_Cataract	CL_Well	CL_MK	CL_PostMK_Visual Loss	Glasses_Well	Dead
Well_ICL	0.98029	0.01	0.005	0.003	0	0	0	0	0.00171
ICL_Complication	0.8	0.16829	0.02	0.01	0	0	0	0	0.00171
ICL_Explant	0	0	0.01829	0	0	0	0	0.98	0.00171
ICL_Cataract	0	0	0.2	0.19829	0	0	0	0.6	0.00171
CL_Well	0	0	0	0	0.98779	0.0005	0	0.01	0.00171

CL_MK	0	0	0	0	0.89	0.00829	0.1	0	0.00171
CL_PostMK_Visual Loss	0	0	0	0	0	0	0.99829	0	0.00171
Glasses_Well	0.01	0	0	0	0	0	0	0.98829	0.00171
Dead	0	0	0	0	0	0	0	0	1
**Year 26_Age 50**									
	Well_ICL	ICL_Complication	ICL_Explant	ICL_Cataract	CL_Well	CL_MK	CL_PostMK_Visual Loss	Glasses_Well	Dead
Well_ICL	0.975319	0.01	0.005	0.008	0	0	0	0	0.001681
ICL_Complication	0.8	0.168319	0.02	0.01	0	0	0	0	0.001681
ICL_Explant	0	0	0.018319	0	0	0	0	0.98	0.001681
ICL_Cataract	0	0	0.2	0.198319	0	0	0	0.6	0.001681
CL_Well	0	0	0	0	0.987819	0.0005	0	0.01	0.001681
CL_MK	0	0	0	0	0.89	0.008319	0.1	0	0.001681
CL_PostMK_Visual Loss	0	0	0	0	0	0	0.998319	0	0.001681
Glasses_Well	0.01	0	0	0	0	0	0	0.988319	0.001681
Dead	0	0	0	0	0	0	0	0	1

Supplementary Table S2. Base case utilities and relevant adjustments.

**State Utilities**				
State	Utility	Range_low	Range_high	Sources
Well_ICL	0.89	0.87	0.91	(Brown et al., 2003; Ieong et al., 2010; Packer, 2016; Pesudovs et al., 2006; Queirós et al., 2012; Syah et al., 2021; van Hemert et al., 2025)
ICL_Complication	0.7	0.5	0.85	
ICL_Explant	0.86	0.83	0.89	
ICL_Cataract	0.6	0.4	0.8	
CL_Well	0.87	0.85	0.9	
CL_MK	0.4	0.2	0.8	
CL_PostMK_VisualLoss	0.6	0.4	0.8	
Glasses_Well	0.88	0.8	0.95	
Dead	0	0	0	
**Subgroup_utility_adjustments**				
Subgroup	Adjust ICL	Adjust CL	Glasses	
High Myopia	0	0	0	**Assumptions** Toric ICL often gives superior uncorrected vision vs CL, Slightly lower post operation satisfaction reported in some hyperopic cohorts and Mixed evidence; conservative adjustments
High Myopia + Astigmatism	0.02	-0.01	0	
High Hyperopia	-0.01	0	0	
High Hyperopia + Astigmatism	0	-0.01	0	
**Subgroup_cost_adjustments**				
Subgroup	ICL_cost_factor	ICL_complication multiplier	cataract_add per_year	**Assumptions** Slightly higher complication risk due to axial length,Toric lens extra cost; modestly higher reposition risk, Higher cataract risk observed in hyperopic ICL cohorts and Combined effects: toric cost + higher cataract risk
High Myopia	1	1.1	0	
High Myopia + Astigmatism	1.2	1.15	0	
High Hyperopia	1.1	1.05	0.002	
High Hyperopia + Astigmatism	1.25	1.15	0.003	
